# Poster Session I - A67 ENDOSCOPIC POLYP SIZE MEASUREMENT USING A LASER-BASED SIZING PROBE: A PILOT STUDY

**DOI:** 10.1093/jcag/gwaf042.067

**Published:** 2026-02-13

**Authors:** M Mahdadi, É Cristea, M Oleksiw, P Aleksieva, V Michal, R Battat, R Djinbachian, C Gefflot, D C Daoud, S Bouchard, M Bouin, J Liu, P Benoit, D Von Renteln

**Affiliations:** Gastroenterology Department, Centre de Recherche du Centre Hospitalier de l’Universite de Montreal, Montreal, QC, Canada; Gastroenterology Department, Centre de Recherche du Centre Hospitalier de l’Universite de Montreal, Montreal, QC, Canada; Gastroenterology Department, Centre de Recherche du Centre Hospitalier de l’Universite de Montreal, Montreal, QC, Canada; Gastroenterology Department, Centre de Recherche du Centre Hospitalier de l’Universite de Montreal, Montreal, QC, Canada; Gastroenterology Department, Centre de Recherche du Centre Hospitalier de l’Universite de Montreal, Montreal, QC, Canada; Centre Hospitalier de l’Universite de Montreal, Montreal, QC, Canada; Centre Hospitalier de l’Universite de Montreal, Montreal, QC, Canada; Gastroenterology Department, Centre de Recherche du Centre Hospitalier de l’Universite de Montreal, Montreal, QC, Canada; Centre Hospitalier de l’Universite de Montreal, Montreal, QC, Canada; Centre Hospitalier de l’Universite de Montreal, Montreal, QC, Canada; Centre Hospitalier de l’Universite de Montreal, Montreal, QC, Canada; Centre Hospitalier de l’Universite de Montreal, Montreal, QC, Canada; Centre Hospitalier de l’Universite de Montreal, Montreal, QC, Canada; Gastroenterology Department, Centre de Recherche du Centre Hospitalier de l’Universite de Montreal, Montreal, QC, Canada

## Abstract

**Background:**

Accurate size measurement of colorectal polyps is important for planning polyp resection and post-polypectomy surveillance.

**Aims:**

This study evaluated a through-the-scope laser-based probe for polyp size measurement during colonoscopies.

**Methods:**

A prospective pilot study was conducted at CHUM in which polyps in enrolled patients were attempted to be measured with a laser-based probe. The primary outcome was the accuracy of laser-based polyp measurements, assessed as mean bias using microscopic sizing as the reference standard. Secondary outcomes included agreement with the reference standard, technical success rate, reasons for failed measurement, and per-polyp measurement time.

**Results:**

Among 323 patients, laser-based measurement was attempted for 237 polyps; 149 had a microscopic size measurement. The technical success rate of the laser-based probe was 40.9% (97/237; 95% CI, 34.6%–47.5%), with mean time per measurement of 1:35. Accounting for specimens that could not be resected en bloc for microscopic measurements, a total of 41/149 (27.5%; 95% CI, 20.7%–35.5%) had paired laser-based and microscopy measurements for the primary analysis. Laser-based measurement demonstrated accurate sizing with minimal bias (–0.10 mm, 95% CI –0.30 to 0.10; P = 0.328) and met the 0.4 mm non-inferiority margin. Agreement with the reference was high (ICC = 0.91; 95% CI, 0.83–0.95), with near-perfect categorical concordance (kappa = 0.85) and 95.1% accuracy (39/41; 95% CI, 82.2–99.2). Bland–Altman limits of agreement ranged from –1.34 mm to 1.14 mm.

**Conclusions:**

Laser-based measurement provided accurate polyp sizing when obtained. However, the technical success rate was limited by longer measurement times and device issues.

**Funding Agencies:**

None

